# Olfactory modulation of stress-response neural circuits

**DOI:** 10.1038/s12276-023-01048-3

**Published:** 2023-08-01

**Authors:** Min-Gi Shin, Yiseul Bae, Ramsha Afzal, Kunio Kondoh, Eun Jeong Lee

**Affiliations:** 1 Department of Brain Science, Ajou University School of Medicine, Suwon, 16499 Korea; 2 AI-Superconvergence KIURI Translational Research Center, Ajou University School of Medicine, Suwon, 16499 Korea; 3 Division of Endocrinology and Metabolism, Department of Homeostatic Regulation, National Institute for Physiological Sciences, National Institutes of Natural Sciences, Okazaki, Aichi 444-8585 Japan; 4 Japan Science and Technology Agency, PRESTO, Okazaki, Aichi 444-8585 Japan

**Keywords:** Stress and resilience, Olfactory system

## Abstract

Stress responses, which are crucial for survival, are evolutionally conserved throughout the animal kingdom. The most common endocrine axis among stress responses is that triggered by corticotropin-releasing hormone neurons (CRHNs) in the hypothalamus. Signals of various stressors are detected by different sensory systems and relayed through individual neural circuits that converge on hypothalamic CRHNs to initiate common stress hormone responses. To investigate the neurocircuitry mechanisms underlying stress hormone responses induced by a variety of stressors, researchers have recently developed new approaches employing retrograde transsynaptic viral tracers, providing a wealth of information about various types of neural circuits that control the activity of CRHNs in response to stress stimuli. Here, we review earlier and more recent findings on the stress neurocircuits that converge on CRHNs, focusing particularly on olfactory systems that excite or suppress the activities of CRHNs and lead to the initiation of stress responses. Because smells are arguably the most important signals that enable animals to properly cope with environmental changes and survive, unveiling the regulatory mechanisms by which smells control stress responses would provide broad insight into how stress-related environmental cues are perceived in the animal brain.

## Stress: The nervous systems responding to smell

Research into the mechanisms through which the sense of smell controls stress responses provides insights into how stress-related environmental cues are perceived in the brain. Smells are important signals enabling animals to sense and cope with environmental changes. A major nervous system associated with stress responses involves corticotropin-releasing hormone neurons (CRHNs) in the hypothalamus. Eun Jeong Lee and colleagues at Ajou University, Suwon, South Korea, and Kunio Kondoh at the National Institute of Natural Sciences in Okazaki, Japan, review what is known about neural circuits influencing CRHNs. They focus on olfactory systems exciting or suppressing the activities of CRHNs that can initiate stress responses. New insights into the mechanisms involved could help understand and perhaps control animal behavior, with potential clinical applications for treating stress in humans.

## Introduction

### Corticotropin-releasing hormone neurons: master controllers of neuroendocrine responses to stress

One of the most common biomarkers of stress is activation of the hypothalamic–pituitary–adrenal (HPA) axis^1^. Activation of the HPA axis is initiated by the release of corticotropin-releasing hormone (CRH) from CRH neurons (CRHNs) in the paraventricular nucleus of the hypothalamus (PVN), followed by a surge in the release of adrenocorticotropic hormone (ACTH) from the pituitary that then induces the secretion of cortisol (humans) or corticosterone (rodents) from the adrenal gland. In rodents, the HPA axis can be activated by a variety of external and internal stressors, including physical stress, such as injury, and psychological stresses associated with predator odors and physical restraint, among others. This suggests that signals from neurons activated in different areas by different stressors should converge on CRHNs, raising many questions about the mechanisms through which different stressors activate the same neurons (i.e., CRHNs). Do different stressors activate different neurons upstream of CRHNs, or do they send signals to CRHNs via the same upstream neurons? What signaling molecules are released from upstream neurons to communicate with CRHNs under different stressful conditions? Answering these questions requires information on the anatomical, molecular, and functional properties of upstream neurons connected to CRHNs clustered in the PVN. Here, we review recent efforts to identify the anatomical locations of neurons upstream of CRHNs, their molecular signatures, and their functions in response to different acute stressors. We then discuss neural circuits that convey olfactory signals to CRHNs and induce or antagonize activation of the HPA axis as one model of the complicated regulation of stress responses.

## Main text

### Stress neurocircuits that converge on CRHNs

#### Classical studies on neuroanatomical inputs to CRHNs

Classically, inputs to the PVN, where CRHNs are clustered, have been investigated by electrical or pharmacological intervention in the function of targeted brain areas or using classical retrograde/anterograde tracers that are capable of being transported from the injected brain area to connected brain areas. For example, injecting the retrograde tracer horseradish peroxidase (HRP) into the PVN area of rats has enabled the identification of a number of brain areas that send projections to the PVN^2–4^. Although classical tracers cannot distinguish CRHNs from other types of neurons in the PVN, they have provided significant insights into brain areas that control the HPA axis, especially when combined with functional studies, as described below.

##### Hypothalamus

**Fig. 1:**
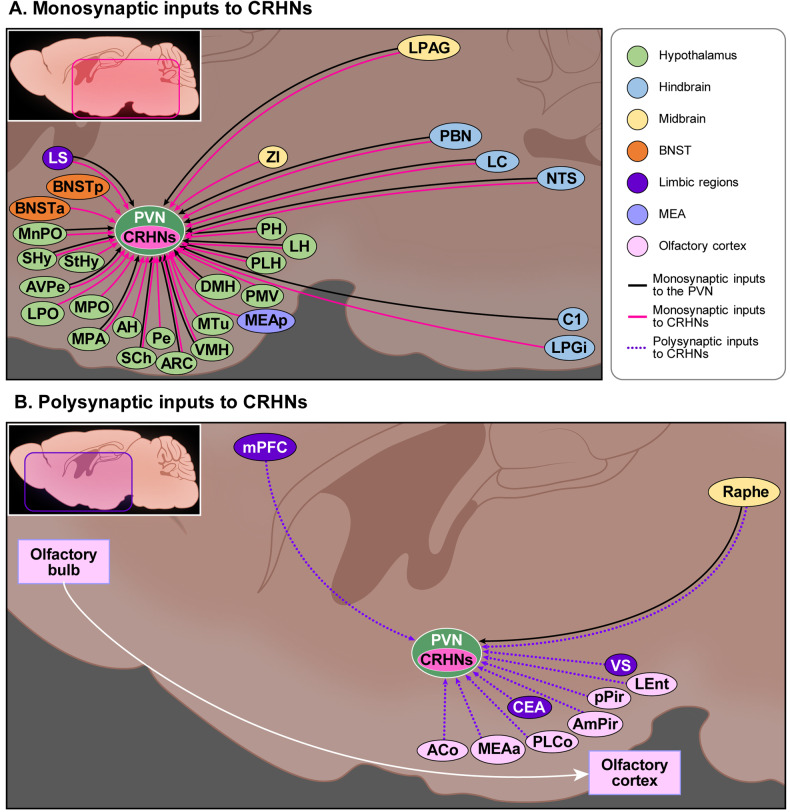
Anatomical inputs to CRHNs revealed by classical studies and transsynaptic viral tracers. Both classical studies and transsynaptic viral tracer-based approaches have revealed many brain regions that have anatomical inputs to CRHNs, either directly (monosynaptic) or indirectly (polysynaptic). While classical studies cannot distinguish CRHNs from other types of neurons in the PVN, Cre-dependent transsynaptic viral tracers can specifically define regions upstream of CRHNs using CRH-Cre mice. **a** Monosynaptic neurons upstream of CRHNs are located in multiple subregions of the hypothalamus, hindbrain, midbrain, and BNST (bed nucleus of the stria terminalis). One area of the limbic regions and one area of the MEA (medial amygdala) also have monosynaptic neurons upstream of CRHNs (LS, lateral septum; BNSTa, bed nucleus of the stria terminalis, anterior part; BNSTp, bed nucleus of the stria terminalis, posterior part; MnPO, median preoptic nucleus; SHy, septohypothalamic nucleus; StHy, striohypothalamic nucleus; LPO, lateral preoptic area; MPO, medial preoptic nucleus; AVPe, anteroventral periventricular nucleus; MPA, medial preoptic area; AH, anterior hypothalamic area; SCh, suprachiasmatic nucleus; Pe, periventricular nucleus of the hypothalamus; ARC, arcuate hypothalamic nucleus; VMH, ventromedial hypothalamic nucleus; DMH, dorsomedial hypothalamic nucleus; MTu, medial tuberal nucleus; PLH, peduncular part of lateral hypothalamus; PMV, premammillary nucleus, ventral part; PH, posterior hypothalamic nucleus; LH, lateral hypothalamic area; MEAp, medial amygdala, posterior part; ZI, zona incerta; LPAG, lateral periaqueductal gray; PBN, parabrachial nucleus; LC, locus coeruleus; NTS, nucleus of the solitary tract; LPGi, lateral paragigantocellular nucleus). **b** Polysynaptic neurons upstream of CRHNs are located in the raphe of the midbrain, as well as limbic regions and the olfactory cortex). Since the olfactory cortex contains polysynaptic neurons upstream of CRHNs, olfactory signals conveyed to the olfactory bulb from the nose can sequentially propagate to the olfactory cortex, intermediate areas, and then to CRHNs (ACo, anterior cortical amygdala; MEAa, medial amygdala, anterior part; PLCo, posterolateral cortical amygdala; AmPir, amygdalo-piriform transition area; pPir, piriform cortex, posterior part; LEnt, lateral entorhinal cortex; CEA, central amygdala; VS, ventral subiculum; mPFC, medial prefrontal cortex).

The PVN, the area of the hypothalamus where most CRHNs reside, receives numerous inputs from neighboring nuclei in the hypothalamus. Classical neuroanatomical studies examining the involvement of these nuclei in stress hormone responses have often revealed mixed features. For example, lesioning of the medial preoptic area (MPA) was shown to abolish the inhibitory effects of testosterone treatment on stress hormone responses^5^, suggesting an inhibitory role of the MPA. However, stimulatory modulation by the MPA has also been reported; in particular, stimulation of a laterally located population was shown to increase stress hormone responses^6^. These results suggest a heterogeneity of MPA neurons that project to the PVN. Similarly mixed findings have been obtained for the dorsomedial hypothalamic nucleus (DMH), with a pharmacological study showing that chemical stimulation of the DMH induces an increase in stress hormones^7^, and other studies reporting the presence of GABAergic inhibitory neurons in the DMH that project to the PVN^8,9^. These results have led to the assumption that the DMH sends both excitatory (presumably glutamatergic) and inhibitory (GABAergic) inputs to the PVN^10^. Consistent with this idea, psychogenic air stress-induced stress hormone responses require activation of the DMH^11^, whereas lesions of the DMH exacerbate stress hormone responses to other psychogenic stresses (elevated place exposure)^12^. The inhibitory role of the DMH in regulating stress hormone responses seems to apply to psychogenic stressors but not systemic stressors (immune challenge). Recent work has provided evidence that the disinhibition of posterior hypothalamic nucleus (PH) neurons inhibits stress hormone increases^13,14^ and that labeled projections of PH neurons in the PVN colocalize with immunostained signals of antibodies against CRH^13^. In addition, a retrograde tracer injected into the PVN was shown to label neurons activated by restraint or a loud noise, not only in the PH but also in the median preoptic nucleus (MnPO)^14^. The suprachiasmatic nucleus (SCh) is also known to send direct inputs to CRHNs in the PVN and play a crucial role in generating the circadian feature of stress hormone release at baseline (i.e., without stressors)^15^. It was previously shown that arginine vasopressin (AVP) released from the SCh inhibits stress hormone responses; more recently, it was also reported that the connection between vasoactive intestinal peptide (VIP)-producing SCh neurons and CRH-producing PVN neurons is crucial for the rhythmicity of stress hormone release^16–18^. The arcuate hypothalamic nucleus (ARC), which acts as the hub of appetite balance regulation by virtue of its populations of both orexigenic (AgRP/NPY neurons) and anorexigenic neurons (POMC neurons), is known to have robust projections to the PVN^19,20^. Although lesions of the ARC dysregulate stress hormone responses^21^ and restraint stress induces the activation of ARC POMC neurons in rats^22^, it remains unclear whether this circuit is involved in specific types of acute stressors. In addition to the hypothalamic nuclei described above, other hypothalamic areas, such as the septohypothalamic nucleus (SHy), anteroventral periventricular nucleus (AVPe), lateral hypothalamic area (LH), peduncular part of the lateral hypothalamus (PLH), and ventromedial hypothalamic nucleus (VMH), contain neurons labeled by retrograde tracers injected into the PVN^23^ (Fig. 1). Nevertheless, the roles of each nucleus in regulating stress hormone responses are largely unknown.

##### Hindbrain

The hindbrain, the lower back part of the brain including most of the brainstem, contains multiple nuclei, such as the nucleus of the solitary tract (NTS), parabrachial nucleus (PBN) and locus coeruleus (LC), which are known to engage in reactive responses to systemic stressors. However, some of these nuclei play important roles in stress hormone responses to psychogenic stressors mediated by PVN-projecting neurons.

The NTS in the hindbrain sends heavy glutamatergic inputs to the PVN, preferentially targeting subregions of the PVN that are either catecholaminergic (noradrenergic and adrenergic)^24,25^ or noncatecholaminergic (e.g., glucagon-like peptide-1 [GLP-1])^26^. Although catecholaminergic NTS neurons that project to the PVN also express other neuropeptides, such as prolactin-releasing peptide and neuropeptide Y^27–29^, they do not coexpress GLP-1^30^. Pharmacological lesions of ascending NTS catecholaminergic pathways cause a reduction in the number of activated cells in the PVN and/or decrease the release of stress hormones into the blood induced by systemic stresses that perturb homeostasis (e.g., ether inhalation, cytokine injection, glucose deprivation) but not by psychogenic stresses (e.g., restraint, foot shock, forced swimming)^31–35^. In contrast, GLP‑1 neurons modulate responses to both systemic and psychogenic stressors^36^. Therefore, it was suggested that these two different cell populations are not only molecularly distinct but also functionally distinct^10^.

The PBN is considered the center of pain sensation. PBN glutamatergic projections to the PVN are predominantly observed in the parvocellular subregion, where CRHNs reside^37^. PBN neurons express neuropeptides, such as calcitonin gene-related peptide, neurotensin and CRH, which are presumably excitatory molecules, and are activated by both systemic (e.g., visceral illness) and psychogenic (e.g., restraint) stressors^38^.

The LC, one of the hindbrain regions, contains mostly norepinephrine-producing neurons, which are reported to be activated by a variety of stressors, including psychological and systemic stimuli^39–42^. However, its inputs to PVN neurons are likely indirect, given its sparse direct input to the PVN^25,43^.

Some C1 neurons, located in the hindbrain (medulla oblongata), innervate the PVN and likely contribute to the release of stress hormones in animals physically stressed by systemic administration of the cytokine interleukin-1 (IL-1)^44^. More recently, Abe et al.^45^ also showed that restraint stress and optogenetic activation of C1 neurons increase plasma stress hormones. These results raise the possibility that C1 neurons can activate the HPA axis via their projections to the PVN (Fig. 1).

##### Midbrain

The midbrain, the topmost part of the brainstem, also contains regions with neurons that project to the PVN, namely, the periaqueductal gray (PAG) and raphe nucleus.

The PAG, a midbrain region surrounding the cerebral aqueduct, can be divided into subregions; importantly, the lateral PAG (LPAG) projects robustly to the PVN^46^. LPAG neurons that project to the PVN are glutamatergic (*Vglut2*+), indicating excitatory inputs from the LPAG to the PVN^47^. Indeed, electrical stimulation of the LPAG causes an increase in stress hormones^48^. Notably, multiple stressors can stimulate the activity of LPAG neurons^49–51^.

Neurons in the raphe, most of which are serotonergic, are known to project to many different brain areas, including the region surrounding the PVN or the PVN itself^52–54^, and stimulate increases in stress hormones^54–56^ (Fig. 1).

##### Limbic regions

The involvement of the limbic system in HPA regulation is clear based on a constellation of reports. However, the connection between limbic regions and the PVN is most likely indirect.

The medial amygdala (MEA) and central amygdala (CEA), subregions of the amygdala, are known to activate the HPA axis^1^. For example, lesions of the MEA or CEA diminish stress hormone responses in a stressor-selective manner^57,58^. The stressor specificities of the MEA and CEA could stem from differences in upstream inputs to those two subregions (olfactory inputs to the MEA versus visceral inputs to the CEA)^59,60^, although both subregions also receive excitatory inputs from the same brain region, the basolateral amygdala (BLA)^61^, which is also involved in stress-related responses^62–66^. It was thought that the MEA and CEA could activate stress hormone responses through intermediate players via a disinhibition mechanism. MEA and CEA projections are mostly GABAergic^67^ and are mainly found in peri-PVN areas and other hypothalamic nuclei, such as the DMH, MPA and PH, as well as the bed nucleus of the stria terminalis (BNST), as revealed by a study that combined retrograde tracing and immunohistochemistry/in situ hybridization^13,68^.

In contrast to the amygdala, the hippocampus plays a role in feedback inhibition of the HPA axis^69,70^. For example, lesions of the hippocampus induce the expression of CRH mRNA in the PVN and elevate stress hormone levels in response to certain stressors^69,71^. More specifically, the ventral subiculum (VS) has been implicated in inhibiting the activities of PVN CRHNs^72,73^. Inputs from the VS to the PVN seem to be indirect, suggesting that the inhibitory actions of the hippocampus on stress hormone responses are mediated by intermediate regions, such as the BNST, MPA, DM and PH, which receive projections from the VS and send projections to the PVN^13,51,74^.

Lesion studies have demonstrated the involvement of the medial prefrontal cortex (mPFC) in the suppression of stress hormone release after stress exposure^75,76^. Similar to the case for the hippocampus, mPFC neurons do not directly project to the PVN but instead project to many other brain regions that have been shown to have projections to the PVN, suggesting that the influence of the mPFC on the HPA axis is mediated by other indirect inputs to the PVN^77^. Given that the hippocampus and mPFC have strong connections and are involved in cognitive function, these neurons may be predominantly involved in psychogenic stress responses.

Other limbic sites, such as the lateral septum (LS), are also known to be involved in regulating the HPA axis. For example, the LS inhibits stress hormone responses to stressors^78^, an effect that might be mediated by its strong connection to the PVN^3,43,79^ (Fig. 1).

##### The bed nucleus of the stria terminalis (BNST)

The BNST is involved in the regulation of the HPA axis; however, the direction of its action varies distinctly according to the BNST subregion, reflecting the very high molecular and functional heterogeneity and anatomical complexity of this region^80^. In particular, two major parts of the BNST—the anteromedial divisions of the BNST (BNSTa) and posterior nuclei (BNSTp)—play distinct roles in regulating stress hormone responses, with lesion studies demonstrating that the former elevates stress hormone responses, whereas the latter attenuates stress hormone release^81^ (Fig. 1). These two BNST subregions are not only functionally distinct but also anatomically distinct in terms of their connections with other regions. For example, the BNSTa receives extensive innervations from the CEA, whereas the BNSTp receives projections from the MEA^82^, although some BNSTa neurons receive inputs from both the hippocampus and mPFC as well^71^. Moreover, the BNSTa receives inputs from both catecholaminergic and GLP-1ergic NTS neurons^26,30,31^, each of which is responsive to different kinds of stressors, as described above.

#### Anatomical mapping of neurons upstream of CRHNs using transsynaptic viral tracers

The PVN contains multiple types of neurons that are involved in different physiological functions. Therefore, although neurophysiological and neuroanatomical studies highlighted in the previous section have provided numerous insights into interactions between the PVN and other brain regions, whether these interactions actually represent direct/indirect synaptic connections to CRHNs or to other cell types in the PVN had remained largely unknown. Furthermore, knowledge on the signaling molecules that mediate signal transmission from these upstream neurons to CRHNs is largely lacking.

**Fig. 2:**
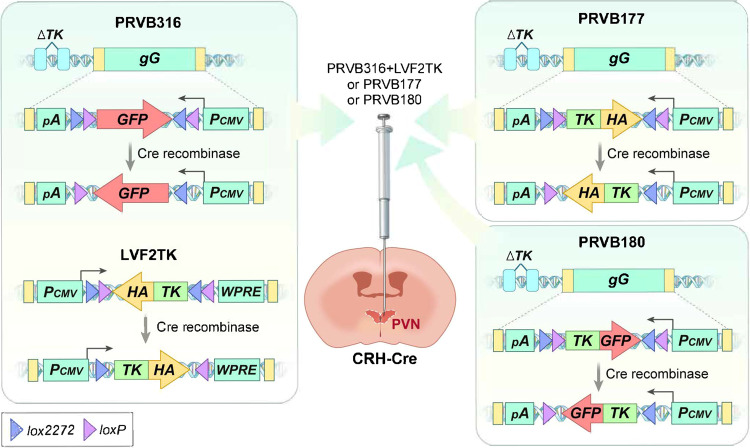
PRV-based retrograde monosynaptic and polysynaptic tracing systems for studying neural circuits upstream of CRHNs. (Left) Monosynaptic tracing system with PRVB316 and LVF2TK. PRVB316 lacks thymidine kinase (TK), which is required for viral replication and spread, but expresses GFP in a Cre recombinase-dependent manner. The lentiviral vector LVF2TK expresses TK in the presence of Cre. PRVB316 can replicate only in Cre+ and LVF2TK-infected cells (because of the exogenous TK expression by LVF2TK) and then travels to directly upstream neurons that do not express TK. In this manner, coinfection of PRV316 and LVF2TK into the PVN of CRH-Cre mice causes PRV infections in brain regions that are directly upstream of CRHNs. (Right) Polysynaptic tracing system with PRVB177 or PRVB180. These two PRVs are able to express HA-tagged (PRVB177) or GFP-fused (PRVB180) TK irreversibly once they infect Cre-expressing cells. Thus, they can sequentially travel across multiple synapses in a time-dependent manner, enabling them to be used as an alternative way to identify neurons that are directly upstream of CRHNs.

To overcome the limitations of previous studies, Buck and colleagues applied retrograde viral tracers that travel across synapses^83,84^ to identify the anatomical locations of upstream neurons that are actually connected to CRHNs^85^. They developed three viral tracers based on the Bartha strain of the pseudorabies virus (PRV), which can cross synapses in a retrograde manner (Fig. 2). Two of these tracer PRVs, PRVB177 and PRVB180, are ‘polysynaptic PRVs’ that lack an endogenous thymidine kinase (TK) gene (which is required for viral replication and spread) but irreversibly express TK upon infection of Cre-expressing cells. Thus, they can travel sequentially across multiple synapses from Cre-expressing cells in a time-dependent manner. PRVB316 lacks an endogenous TK gene, even in the presence of Cre, but functions as a ‘monosynaptic PRV’ when coinfected with a lentivirus (LVF2TK) expressing TK in the presence of Cre recombinase. In other words, PRVB316 can replicate in TK+ neurons by virtue of coinfection with LVF2TK and crosses only one synapse.

By injecting poly- or monosynaptic PRVs into Cre-expressing CRHNs of CRH-Cre mice, Buck and colleagues identified neurons that are either directly or indirectly connected to CRHNs^85^ (Fig. 1). A total of 31 brain areas were infected with monosynaptic PRVs (PRVB316), most of which were also infected with polysynaptic PRVs (PRVB177 or PRVB180) on day 3 after PRV infection, suggesting that it takes ~3 days for polysynaptic PRVs to label presynaptic neurons that directly connect to starting CRHNs. Among the brain areas exhibiting monosynaptic PRV-infected neurons as well as polysynaptic PRV-infected neurons 3 days after virus injection were 18 hypothalamic nuclei, two midbrain regions, three hindbrain regions, and three other areas. This previous study generated a list of all hypothalamic nuclei that are directly upstream of CRHNs and cannot be investigated easily through lesion or pharmacological manipulation owing to their proximity to PVN CRHNs. In addition to hypothalamic nuclei, many areas known to regulate stress hormone responses through projections to the PVN were revealed to have monosynaptic or polysynaptic connections upstream of CRHNs. For example, the PBN and NTS in the hindbrain were shown to be directly upstream of CRHNs.

This study also identified a previously unexpected area that could control CRHNs or the HPA axis. Although it was previously suggested that the lateral paragigantocellular nucleus (LPGi) sends projections to the PVN^23^, Kondoh et al.^85^ were the first to show that the LPGi is actually directly upstream of CRHNs (Fig. 1). The LPGi, located in the rostral ventrolateral medulla (RVLM) of the hindbrain, is known to be involved in autonomic system regulation, including regulation of blood pressure^86^. Because the autonomic system controls stress responses through the sympathetic nervous system (SNS), these findings suggest the interesting possibility that the LPGi is involved in stress responses through both the HPA axis and the SNS. A subsequent report demonstrated that the LPGi is responsive to predator odor stress^87^ (see below).

Consistent with the previous idea that the VS is indirectly upstream of the PVN, it has been shown that only polysynaptic PRVs and not monosynaptic PRVs infect neurons in the VS. In contrast to the previously held view that the MEA or LC sends inputs to the PVN indirectly, monosynaptic PRV infection was found in the posterior part of the MEA (MEAp) and LC (Fig. 1). Additionally, MEA neurons monosynaptic to CRHNs are both glutamatergic and GABAergic, a finding that contrasts with earlier thinking that MEA projections to the PVN through intermediate brain regions such as the BNST or other hypothalamic nuclei are extensively GABAergic^67^.

It has been further shown that PVN CRHNs receive direct synaptic inputs from the zona incerta (ZI), which is located in a subthalamic region and is known to have extensive connections to other brain regions (Fig. 1). It was recently reported that among the diverse roles of the ZI is the regulation of behaviors related to stress and fear^88^. Although the role of the ZI in regulating the HPA axis has remained elusive, connections between the ZI and CRHNs could mediate a subset of stress responses. The types of stressors that influence ZI neurons and the mechanisms through which the ZI modulates stress hormone responses are still unclear.

Taking advantage of the availability of mono- and polysynaptic PRVs, Kondoh et al.^85^ defined the connectivity structure between olfactory regions and CRHNs. In olfactory areas, several olfactory cortex (OC) regions that receive olfactory information from the main olfactory epithelium contained only polysynaptic PRV-infected neurons, but not monosynaptic PRV+ neurons, 4 days after injection, suggesting that the OC contains neurons that are indirectly upstream of CRHNs. PRV-infected neurons were detected in five OC areas, including the posterior part of the piriform cortex (pPir), anterior cortical amygdala (ACo), posterolateral cortical amygdala (PLCo), amygdalo-piriform transition area (AmPir), and lateral entorhinal cortex (LEnt) (Fig. 1). The vomeronasal amygdala (VA), an area downstream of the vomeronasal organ, was also shown to contain polysynaptic PRV-infected neurons, suggesting that these areas can convey signals to CRHNs. In contrast to the OC, one VA area—the MEAp—contained monosynaptic PRV-infected neurons, indicating that this area is directly connected to CRHNs. These results suggest that multiple olfactory areas can stimulate CRHNs either directly or indirectly by transmitting stressful olfactory cues, such as predator odors (see below).

#### Molecular mapping of neurons upstream of CRHNs

To superimpose a molecular map on the anatomical map of neurons upstream of CRHNs, Buck and colleagues developed two complimentary methods: receptor-assisted mapping of upstream neurons (RAMUN)^87^ and single-cell RNA sequencing (scRNA-seq) of upstream neurons, the latter of which was termed Connect-seq^89^. The RAMUN procedure consists of two steps. The first step identifies neurotransmitter or neuromodulator receptors expressed in CRHNs using a scRNA-seq method, revealing the entire repertoire of neurotransmitter and/or neuromodulator signaling received by CRHNs. The second step identifies brain areas that transmit specific neurotransmitters or neuromodulators to CRHNs by determining the locations of neurons upstream of CRHNs that express specific neurotransmitter or neuromodulator molecules known to bind to receptors expressed in CRHNs. In these experiments, upstream neurons are labeled by infecting CRH-Cre mice with the Cre-dependent retrograde viral tracer PRVB177, after which brain sections are prepared from these animals and costained with antibodies for hemagglutinin (HA), a PRVB177 marker (Fig. 2), and with riboprobes for markers of individual ligands (e.g., transporters or biosynthetic enzymes) indicative of their expression. Connect-seq takes an alternative approach, first investigating the repertoire of neurotransmitter or neuromodulator molecules expressed in upstream neurons of CRHNs. This is accomplished by injecting the Cre-dependent polysynaptic PRV PRVB180, expressing green fluorescent protein (GFP)-fused TK, into CRH-Cre mice and then isolating monosynaptically connected neurons, which should express GFP-TK 3 days after infection, by fluorescence-activated cell sorting (FACS). Single GFP+ neurons are collected into individual wells of 96-well plates, after which neurotransmitter or neuromodulator molecules expressed in individual isolated neurons are analyzed by scRNA-seq. The locations of neurons that express specific neurotransmitters or neuromodulators are then identified by in situ hybridization in brain sections of CRH-Cre mice infected with PRVs.

The first part of the RAMUN method, in which scRNA-seq was performed on isolated CRHNs, revealed that a large variety of neurotransmitter and neuromodulator receptors are expressed in CRHNs, including 41 ligand-gated ion channels that bind to fast neurotransmitters such as glutamate, GABA, glycine, acetylcholine, and adenosine triphosphate (ATP). In addition, it showed that a total of 63G protein-coupled receptors (GPCRs) for a number of neurotransmitters, biogenic amines, and/or neuropeptides were expressed in at least 2 of a total of 18 CRHNs. In addition to these 63 GPCRs, 10 other GPCRs were detected in just one CRHN (Table 1). Previous scRNA-seq data obtained from neurons located in hypothalamic regions broader than the PVN^90,91^, available in the Gene Expression Omnibus (GSE74672), revealed the expression of 80 GPCRs for neurotransmitters, biogenic amines and/or neuropeptides in at least 2 of a total of 86 CRH+ cells, and 54 of 89 GPCRs were found in common in both datasets. In another very recent study in which scRNA-seq data were obtained for 706 PVN neurons^92,93^, we found that at least 2 of 60 CRH + PVN cells expressed 43 GPCRs for neurotransmitters, biogenic amines, and/or neuropeptides. Together, three independent scRNA-seq datasets showed that CRHNs express a total of 105 GPCRs for neurotransmitters, biogenic amines and/or neuropeptides, 90 of which were detected in at least two CRH+ cells in either dataset. A list of gene names for GPCRs detected in three independent studies is presented in Table 1. Taken together, these observations suggest that a large variety of neurosignaling molecules released by neurons upstream of CRHNs can modulate CRHN excitability in response to different types of stressors by binding to their cognate receptors expressed in CRHNs.

**Table 1:** GPCR ligands in CRHNs .

Among the GPCRs expressed in CRHNs, those identified by three independent transcriptome analyses of single CRH + PVN neurons (Lee et al. [GSE143135; 18 cells], Xu et al. [GSE148568; 60 cells], and Romanov et al. [GSE74672; 86 cells]) are those for a variety of neuropeptides, neurotransmitters (glutamate, GABA, glycine, acetylcholine, adenosine, or ADP/ATP) and biogenic amines (epinephrine, norepinephrine, dopamine, histamine, and serotonin). Sequencing analyses of single neurons upstream of CRHNs (“Connect-seq”) revealed that all ligands (except melanin-concentrating hormone) were expressed in at least one of 117 hypothalamic neurons upstream of CRHNs. Pmch+ neurons upstream of CRHNs might be located in other brain regions. Although some receptors were expressed in only one cell out of a total of 126 CRH+ cells (red), all receptors (except GNRHR) for the ligands detected by Connect-seq were also found in at least one CRH+ cell. This table shows how applying sequencing analyses to both receptors in downstream neurons (e.g., CRHNs) and ligands in upstream neurons is a powerful approach for providing the genetic information necessary to dissect specific circuits at the molecular level.

**Fig. 3:**
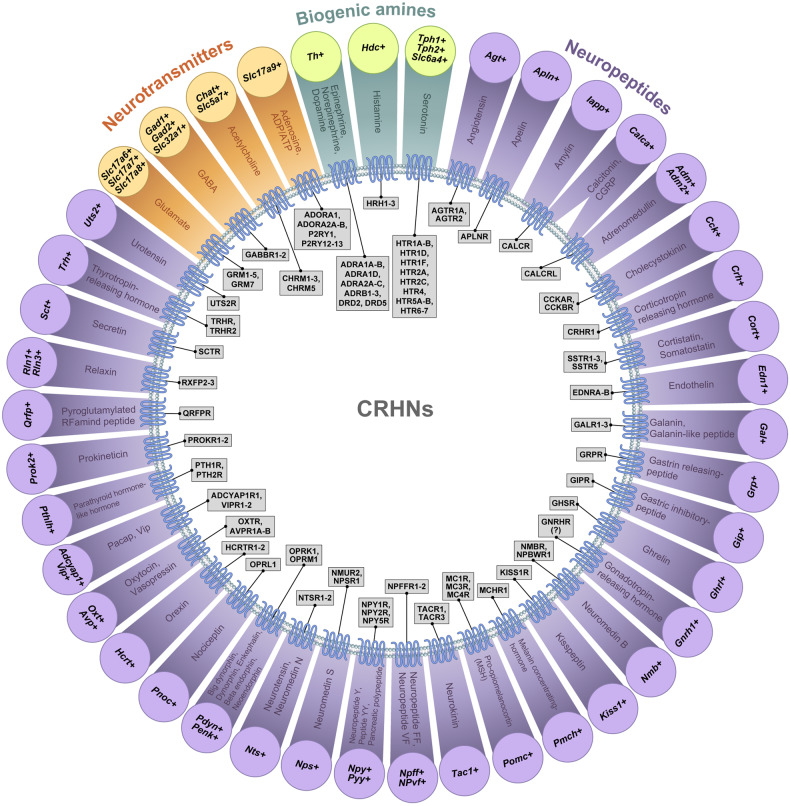
Repertoire of neurosignaling molecules that can be released from upstream neurons to control CRHN activity. The combinations of ligands, marker genes for ligand-expressing cells, and receptors expressed in CRHNs are summarized. The results obtained from three independent scRNA-seq datasets (GSE143135, GSE74672 and GSE148568) indicate that CRHNs express at least 105 GPCRs for neurotransmitters, biogenic amines, and/or neuropeptides. Studies performed using the Connect-seq method provide evidence that ligands for most of these receptors are actually expressed in neurons upstream of CRHNs. These results suggest that a large variety of neurosignaling molecules released by neurons upstream of CRHNs can modulate CRHN excitability by binding to their cognate receptors in CRHNs in response to different kinds of stressors.

To explore whether receptors on CRHNs identified by scRNA-seq actually receive neurosignaling molecules from neurons upstream of CRHNs, we analyzed the expression of neurosignaling molecules in CRHN-upstream neurons by costaining brain sections for PRV (neurons upstream of CRHNs) and genes specific for neurosignaling molecules (the second step in RAMUN) or by scRNA-seq analysis of PRV+ neurons upstream of CRHNs (Connect-seq). Most neurosignaling molecules that are known to be ligands of CRHN receptors identified by scRNA-seq analysis of CRHNs were also detected by Connect-seq, as shown in Table 1 and Fig. 3. These results suggest that combining receptor profiling of downstream neurons with ligand profiling of upstream neurons using scRNA-seq is an effective strategy for identifying neurosignaling molecules involved in communication between two groups of neurons.

**Fig. 4:**
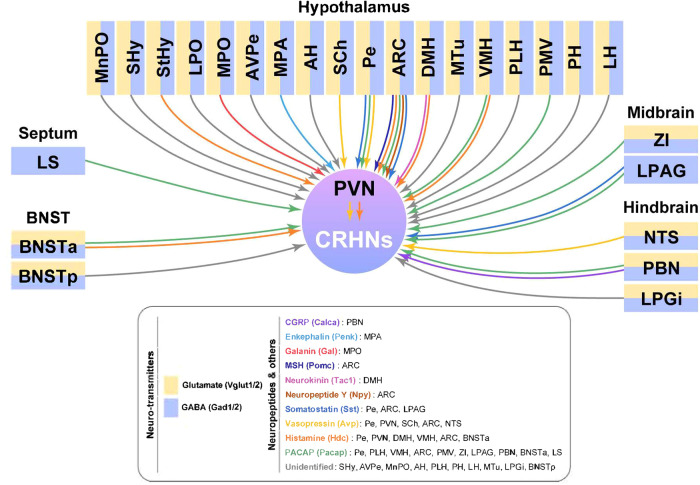
Molecular map superimposed on the anatomical circuit map of neurons upstream of CRHNs. The anatomical locations and types of neurosignaling molecules expressed in neurons upstream of CRHNs, as reported by Hanchate et al., are summarized. The colors under each brain area represent the neurotransmitter expressed in neurons upstream of CRHNs (yellow, glutamate; blue, GABA). The colors of arrows to CRHNs represent the types of neuropeptide signaling from each specific brain area. Both glutamatergic (*Vglut1/2*+) and GABAergic (*Gad1/2*+) PRV+ neurons are located in most upstream brain areas, suggesting that CRHNs, which express a number of receptors for glutamate or GABA (Table 1), can receive either excitatory or inhibitory signals from their upstream regions. Unlike glutamate and GABA, neuropeptides that are transmitted to CRHNs could be released from specific brain regions because the location of upstream neurons expressing neuropeptide ligands of CRHN receptors is selective.

scRNA-seq data from dissociated single cells should be verified by staining for proteins or mRNAs of identified genes to establish the original location of the cells. Surprisingly, costaining for PRV and marker genes of neurosignaling molecules identified by both RAMUN and Connect-seq revealed that CRHNs receive both excitatory (glutamatergic) and inhibitory (GABAergic) inputs without a distinguishable bias. Furthermore, both glutamatergic (*Vglut1/2*+) and GABAergic (*Gad1/2*+) PRV+ neurons are located in most upstream brain areas, suggesting that CRHNs, which express a number of receptors for glutamate or GABA (Table 1), receive both excitatory and inhibitory signals from their upstream regions. This could also explain inconsistent results from previous reports lacking cell type analyses, such as the mixed properties of the DMH in regulating stress hormone responses^7–9^. Unlike for glutamate- and GABA-expressing neurons, the location of upstream neurons expressing neuropeptide ligands for CRHN receptors is selective. Figure 4 shows a summary of neurosignaling molecules in CRHN-upstream neurons in specific brain regions that were validated by costaining for PRV and riboprobes for marker genes of the neurosignaling molecules. Interestingly, different types of neuropeptides were sometimes detected in single PRV+ (i.e., upstream) neurons in some brain regions upstream of CRHNs, such as the ARC^89^. These data indicate that CRHNs can be modulated by different neuropeptides released from different upstream regions. It is also possible that neuropeptides and neurotransmitters act synergistically on CRHNs, an idea supported by data from Connect-seq showing that some upstream neurons coexpress neuropeptides together with glutamate or GABA^89^. Taken together, these findings establish the molecular signatures of neurons upstream of CRHNs, which can be used for parsing which neurosignaling molecules released from upstream neurons in different brain areas regulate stress hormone cascades in response to different kinds of stress.

### Neurocircuits of stress and their blockage by odors

#### Neuroanatomical studies of stress induced by predator odors

Having elucidated neural circuits governing stress hormone responses, the next aspect that needs to be considered is how specific stress stimuli are transmitted or regulated to activate CRHNs through the previously described neural circuits. Olfactory stressors such as predator odors evoke innate responses, including behavioral reactions (e.g., escape and freezing) and release of stress hormones via HPA axis activation that are critical for animal survival^94,95^. How do such olfactory danger signals activate CRHNs to initiate HPA axis activation? The detection of predator odors starts with the activation of olfactory sensory neurons in the main olfactory epithelium (MOE) and/or the vomeronasal organ (VNO), which send signals to the main olfactory bulb (MOB) and accessory olfactory bulb (AOB), respectively. Whereas the AOB projects to the BNST, the MEA, the posteromedial cortical amygdaloid nucleus (PMCo), and axons of MOB neurons preferentially project to the anterior olfactory nucleus (AON), the anterior and posterior piriform cortex (aPir and pPir), the olfactory tubercle (OT), the LEnt, the MEA, and ACo and PLCo^96^. Thus, one possible idea is that neurons in specific olfactory areas innervated by the MOB or AOB are activated by predator odors, whereupon they transmit signals to CRHNs that cause the release of stress hormones. The MEA, which is upstream of CRHNs, is also reported to be involved in the stress response to predator odor^95,97–99^, as is the MOE. In this latter context, it was reported that predator odor (fox feces odor)-induced stress hormone release is substantially reduced in mice lacking sensory detection in the dorsal MOE, raising the possibility that MOB-projecting olfactory areas, such as the olfactory cortex (Pir, LEnt and PLCo), shown to lie upstream of CRHNs^85^, are involved in stress hormone responses to predator odors.

#### Neurocircuits upstream of CRHNs activated by predator odors

Although neuroanatomical studies have provided insights into how predator odors activate neurons in individual brain areas, it is not known whether activated neurons in these areas are actually upstream of CRHNs. To address this question, Buck and colleagues analyzed the expression of a neuronal activation marker in PRV-infected olfactory cortex neurons upstream of CRHNs^85,87^. Surprisingly, only neurons upstream of CRHNs in AmPir were found to be responsive to two kinds of predator odors, the fox predator odorant 2,5-dihydro-2,4,5-trimethylthiazoline (TMT) and bobcat urine^85^, although several OC areas were shown to contain neurons upstream of CRHNs (see above). Indeed, these neurons contribute to stress hormone increases induced by predator odors, such that activation of neurons in and around the AmPir induced a stress hormone response, whereas silencing of the AmPir dramatically reduced stress hormone responses to predator odors^85^. It was recently reported that an alarm pheromone released by a threatened mouse also activates the AmPir and induces stress hormone responses^100^. Other OC areas containing neurons upstream of CRHNs might play different roles, for example, suppressing CRHNs, an effect that could be attributable to exposure to odors that block stress hormone responses (see below).

Because PRV-infected AmPir neurons appear 4 days after polysynaptic PRV injection into the PVN of CRH-Cre mice and monosynaptic PRVs starting in PVN CRHNs cannot travel to the AmPir^85^, there are likely two synapses upstream of CRHNs. The obvious next question is where are the neurons that transmit predator odor signals from AmPir neurons to CRHNs. To explore this question, Lee et al.^87^ examined the locations of neurons directly upstream of CRHNs that are activated by the predator odorant, TMT. Two areas showed significant TMT-induced activation of upstream neurons: the BNSTa and LPGi. These results suggest that either the BNSTa or LPGi (or both) acts as an intermediate relay between the AmPir and CRHNs. In support of this idea, it has been shown that AmPir neurons send dense projection to the BNSTa but not the LPGi (Kondoh et al., unpublished data), suggesting a possible role of BNSTa neurons in predator odor-mediated stress hormone responses. Further neural tracing analyses could help reveal the brain area that relays signals of predator odor from the AmPir to CRHNs.

Interestingly, the BNSTa shows significant activation of PRV+ neurons not only by TMT but also by restraint, a nonolfactory stressor, whereas LPGi PRV+ neurons are activated only by TMT and not by restraint. In contrast, ARC upstream neurons, including pro-opiomelanocortin (*Pomc*)-positive subsets, are activated by TMT but not by restraint. These results indicate that predator odors can activate neurons upstream of CRHNs in multiple brain areas, some of which are activated more specifically by predator odor (e.g., LPGi), whereas others are responsive to multiple stressors (e.g., BNSTa)^87^.

#### Neuroanatomical studies of blocking stress by odors

Odors are volatile chemicals that can evoke aversive, neutral, or attractive preferences in animals^101–103^. For example, predator-related odors (e.g., TMT) are aversive, whereas food-related odors (e.g., propionic acid [PPA]; Swiss cheese scent) can be attractive to mice^103^. Interestingly, a mixture of odors can change the perception of a single odorant. For example, it has been reported that trimethylamine (TMA) can block TMT-induced aversive behaviors^103^. Furthermore, some odors can alleviate stress hormone responses to stressful odors. Sato and colleagues reported that a rose odor attenuates TMT exposure-induced increases in blood stress hormone levels and neuronal activation in the ventrorostral portion of the ACo and the medial portion of the BNSTa in mice^101^. In contrast, rose odor exposure does not affect TMT-induced neuronal activation in the OB, suggesting that rose odor alleviates stress hormone increases induced by TMT through stress hormone response-related neural circuits downstream of the OB, rather than by masking olfactory detection in the nose or olfactory bulb. This same group also reported that hinokitiol (Hino), a woody scent, but not S(+)-carvone, a caraway odor, blocks TMT-induced stress hormone increases as well as TMT-activated c-Fos expression in both the BNSTa and ACo^101^. Interestingly, because all odors used in these exposure regimens were novel to experimental mice, these results collectively indicate that these animals have innate circuits for odor-mediated stress blocking. However, whether neurons in the ACo or BNSTa that are activated by TMT but blocked by rose odor or Hino are indeed upstream of CRHNs and whether those neurons are required for blocking effects on predator odor-induced stress hormone responses have not yet been investigated.

#### Neurocircuits upstream of CRHNs that mediate the blocking of stress by odor

As part of efforts to explore the neural circuits involved in odor-mediated blocking of stress hormone responses, Lee et al.^104^ identified two odorants—2-phenylethanol (2PE), a rose scent, and trimethylamine (TMA), a fish scent—that block stress hormone responses to three different stressors: physical restraint, predator odor, and male‒male social confrontation. Two other odors tested, the woody scent Hino and cheese scent PPA, had no effect when administered to stressed animals. These results are consistent with the reported ability of rose oil to inhibit the stress hormone response to TMT^101^ but differ from the reported ability of Hino to do so^102^. The discrepancy between the results reported by Lee et al.^104^ and Murakami et al.^102^ could be explained by the concentration used in each study: Lee et al.^104^ used TMT and Hino at the same concentration, whereas Murakami et al.^102^ used a concentration of Hino 50 times higher than that of TMT. The results suggest that there is a particular concentration range required for odor-mediated blocking processes. It should be noted that the same odors can trigger opposing behavioral responses depending on their concentration^103,105^. However, certain odors, such as TMA, can maintain the same valence (attraction) at various concentrations^103^. It was observed that TMA had blocking effects on TMT-induced aversion at all concentrations when it was attractive to mice. Therefore, it can be inferred that the blocking effects of odors may depend on the concentration of the odor, as well as the odor’s valence.

### Odor-mediated blocking of stress can occur via inhibition of the excitatory neurons that send stressor signals to CRHNs

**Fig. 5:**
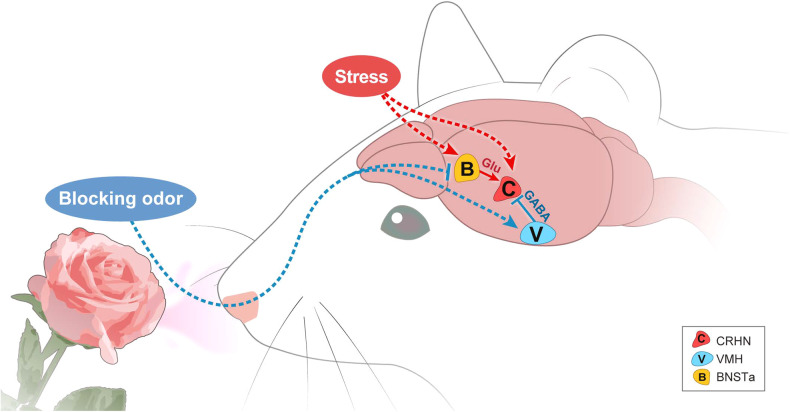
Neurocircuits of odor-mediated blocking effects on stress hormone responses. Signals from blocking odors can travel to the VMH, where they activate inhibitory GABAergic neurons presynaptic to CRHNs and thereby block CRHN activation and stress hormone increases. Odor-mediated blocking signals can also travel to the BNSTa, where they inhibit stressor-induced activation of excitatory glutamatergic neurons upstream of CRHNs and reduce their ability to stimulate CRHNs and stress hormone increases.

How are odors able to block stress hormone responses? Lee et al.^104^ found that such blocking could occur through two different pathways (Fig. 5).

The first possibility is that odors such as those of 2PE and TMA block stress hormone responses by interfering with the activation of neurons upstream of CRHNs induced by multiple stressors; this is conceivable since both compounds are effective in blocking stress hormone increases induced by different stressors. To test this idea, Lee et al.^104^ investigated CRHN-upstream neurons in the BNSTa that were previously reported to be activated by two different stressors: a predator odor (TMT) and restraint^87^. Indeed, both 2PE and TMA dramatically reduced the number of BNSTa neurons upstream of CRHNs activated by subjecting animals to TMT exposure or physical restraint. These results are in line with those of a previous report showing that rose oil inhibits stress hormone responses as well as c-Fos expression in the BNST and ACo^101^. However, Lee et al.^104^ proposed two novel points: (1) smelling a single rose odorant (2PE) exerts blocking effects on stress hormone responses; and (2) specific subsets of excitatory (*Vglut1/2*+) BNSTa neurons upstream of CRHNs (PRV+) are activated by stressors but inhibited by 2PE. Others have previously shown that the BNSTa is important in excitation of the HPA axis in that BNSTa lesions reduce stress hormone responses and inhibit restraint-induced acute activation of PVN neurons^81,106^. It has also been shown that signaling molecules released from the BNSTa to the PVN include glutamate^107^ as well as CRH, which is predominantly an excitatory neuropeptide^108^. Thus, it is also possible that excitatory glutamatergic BNSTa neurons upstream of CRHNs involved in odor-mediated blocking effects also express CRH. These results suggest that the transmission of excitatory stress signals from BNSTa neurons to CRHNs is drastically reduced by a blocking odor, which alleviates the activation of CRHNs by stressors.

### Blocking of stress by odors can occur through activation of inhibitory neurons upstream of CRHNs

Lee et al.^104^ also suggested another mechanism for the odor-mediated blocking of stress hormones: the inhibition of CRHNs through the activation of inhibitory neurons upstream of CRHNs. This mechanism involves upstream GABAergic neurons connected to CRHNs in the ventromedial hypothalamus (VMH). The odors of both 2PE and TMA, but not predator odors, activate VMH inhibitory neurons directly upstream of CRHNs, suggesting the possibility that this activation is specific to blocking odors^87,104^. Indeed, a chemogenetic analysis revealed that VMH GABAergic neurons are required for odor-mediated blocking effects on stress hormone responses, demonstrating that the activation of VMH GABAergic neurons decreases the stress hormone response to a stressor, mimicking odor-mediated blocking effects, and that their silencing prevents the ability of 2PE to block this response. These results suggest that VMH GABAergic neurons inhibit CRHNs directly through the release of GABA.

The VMH can be spatially and functionally separated into a ventrolateral part (VMHvl) that governs aggression and reproductive behaviors and a dorsomedial part (VMHdm) that controls fear and defensive behaviors^109^. The VMHvl contains most GABAergic cell bodies of the VMH, whereas the VMHdm is rich in excitatory neurons. Notably, Choi et al.^110^ showed that reproductive olfactory stimulus-responsive inhibitory neurons in the MEApd project only to the VMHvl and possibly enable reproductive behaviors by inhibiting inhibitory neurons in the VMHvl. Although Lee et al.^104^ did not present the detailed spatial distribution of GABAergic VMH neurons upstream of CRHNs, these findings raise the interesting possibility that 2PE- or TMA-activated GABAergic neurons in the VMH upstream of CRHNs might also be responsive to olfactory reproductive signals, inviting future studies about the possible influence of 2PE and TMA on reproduction-related stress amelioration. In support of this idea, it was shown that preexposure to female urine, a reproductive cue, can block stress hormone responses in male mice, although whether female urine can activate VMHvl inhibitory neurons was not investigated^111^.

It is also conceivable that some stress signals to CRHNs can be modulated by blocking odors in other brain areas beyond the BNSTa or VMH, such as the AmPir, which activates hormonal responses associated with fear, and other OC areas that have neurons upstream of CRHNs. It is also possible that neurons upstream of CRHNs in the BNSTa and VMH interact to modulate the activity of CRHNs. Furthermore, it is worth noting that neurons activated by blocking odors, such as the odors of 2PE and TMA, can be located in different areas depending on the concentration of the odor. This is because the valence of odors and their blocking effects on stress are highly dependent on their concentrations^102–105^.

## Conclusions and future questions

The application of retrograde viral tracer techniques to investigate stress circuits that converge on CRHNs in combination with scRNA-seq provides rich insights into the molecular signatures of neurons upstream of CRHNs. This information can be used to dissect the role of many regions upstream of CRHNs in regulating stress hormone responses as well as other phenotypes (e.g., fear behavior) regulated by CRHNs^92,112,113^. These powerful scRNA-seq–based viral tracing tools can also be extended to other systems (e.g., the dopamine system) beyond the stress hormone system.

Stress hormone responses induced by odors are more dramatic in rodents than in humans, reflecting the fact that rodents have a more sensitive sense of smell owing to their much higher numbers of olfactory receptors in the olfactory epithelium. Interestingly, however, odor-mediated blocking effects on stress hormone responses and the types of blocking odors seem to be evolutionarily conserved from rodents to humans: as is the case in rodents, a rose scent reduces stress hormone levels in stressed humans^114^. Strikingly, the odor of 2PE is also attractive to *Caenorhabditis elegans (C. elegans)*^115^, and we have found that 2PE exerts blocking effects on stress-induced shortening of longevity in *C. elegans* (unpublished data). These observations suggest that the ligand (rose scent) and receptor pair and/or circuits downstream of the sense of smell coevolved in the same direction, causing attractive perception in host organisms^116^. If it is true that humans evolved to be attracted to the rose scent and obtain relief, even in stressful conditions without prior exposure, it would be important to investigate how the rose scent blocks the odor of stress. Moreover, the clinical use of blocking odors, for example, to reduce pain levels by relieving stress responses, could be universally effective in humans regardless of their race, background, and previous experience.

The stress responses discussed in this review are limited to the release of stress hormones and do not encompass other aspects of stress responses, such as those induced by the autonomic nervous system or stress-related behaviors. Furthermore, we did not delve into subsequent stress responses that have clinical implications, such as inflammation, which can be caused by cortisol. It should be noted that the definition of “stress” used in this review is restricted to acute stresses experienced by experimental animals and does not include chronic stress. As a result, further investigations are required to explore the effects of odors on stress responses in terms of multiple aspects induced by different types of stress and varying durations. This would broaden our understanding of the potential clinical and practical uses of odors in mitigating stress.
